# Localization of a bacterial group II intron-encoded protein in human cells

**DOI:** 10.1038/srep12716

**Published:** 2015-08-05

**Authors:** Mercedes Reinoso-Colacio, Fernando Manuel García-Rodríguez, Marta García-Cañadas, Suyapa Amador-Cubero, José Luis García Pérez, Nicolás Toro

**Affiliations:** 1 Grupo de Ecología Genética, Estación Experimental del Zaidín, Consejo Superior de Investigaciones Científicas, Calle Profesor Albareda 1, 18008 Granada, Spain; 2 GENYO (Centro Pfizer-Universidad de Granada-Junta de Andalucía de Genómica e Investigación Oncológica), PTS Granada, Avda. de la Ilustración 114 18016 Granada, Spain

## Abstract

Group II introns are mobile retroelements that self-splice from precursor RNAs to form ribonucleoparticles (RNP), which can invade new specific genomic DNA sites. This specificity can be reprogrammed, for insertion into any desired DNA site, making these introns useful tools for bacterial genetic engineering. However, previous studies have suggested that these elements may function inefficiently in eukaryotes. We investigated the subcellular distribution, in cultured human cells, of the protein encoded by the group II intron RmInt1 (IEP) and several mutants. We created fusions with yellow fluorescent protein (YFP) and with a FLAG epitope. We found that the IEP was localized in the nucleus and nucleolus of the cells. Remarkably, it also accumulated at the periphery of the nuclear matrix. We were also able to identify spliced lariat intron RNA, which co-immunoprecipitated with the IEP, suggesting that functional RmInt1 RNPs can be assembled in cultured human cells.

Mobile group II introns are catalytic RNAs and mobile retroelements that were initially discovered in the mitochondrial and chloroplast genomes of lower eukaryotes and plants^1^ and subsequently identified in bacteria and archaea^2^,^3^,^4^. It has been suggested that the spliceosome^5^ descends from group II introns, but these introns are absent from the protein-coding genes of the nuclear genomes of eukaryotes^6^,^7^,^8^,^9^. Nevertheless, there are several lines of evidence supporting this hypothesis. For example, the RNA-catalyzed splicing reactions are identical for group II introns and snRNAs, which also display structural and functional similarities^5^. It has been speculated that group II introns invaded the eukaryotic lineage at about the same time as endosymbiosis was established between an α-proteobacterium and an archaeal host^8^. Group II introns would thus have played an important role in the evolution of several features of eukaryotic cell organization, such as the endomembrane apparatus, including the nucleus, and the development of nonsense-mediated RNA decay (NMD) and the ubiquitin system. In this scenario, group II introns evolved into more efficient spliceosome-dependent introns^9^,^10^. However, components of telomerase, vertebrate long interspersed elements (LINEs) and most nuclear introns may also be descended from group II introns^6^,^7^.

Group II introns have a conserved secondary structure consisting of six domains (DI to DVI) arranged around a central wheel^11^,^12^,^13^. Most bacterial group II introns have a multifunctional intron-encoded protein (IEP) ORF within DIV^2^. Most IEPs have two conserved domains: an N-terminal reverse transcriptase (RT) domain and domain X, which has been associated with RNA splicing or maturase activity. Some of these IEPs also contain a C-terminal DNA-binding and DNA endonuclease domain^14^. After splicing, the RNA of group II introns forms a ribonucleoprotein particle (RNP), consisting of the IEP and the excised intron RNA lariat. These RNPs can promote intron insertion into DNA target sites, by a mechanism involving the IEP and base-pairing of the intron RNA (exon-binding sites [EBS]) to the target DNA (intron-binding sequences [IBS])^15^. Group II introns have high insertion frequencies and specificities, but the redesign of several introns to insert into a desired DNA target, such as Ll.LtrB from *Lactococcus lactis*^16^, EcI5 from *E. coli*^17^ and RmInt1 from *Sinorhizobium meliloti*^18^,^19^, has been demonstrated. Group II intron-based (Ll.LtrB) gene-targeting methods have also recently been adapted for higher organisms. Integration of the Mg^2+^ -dependent group II intron into plasmid target sites in *Xenopus laevis* oocyte nuclei and *Drosophila* and zebrafish embryos has been shown to be promoted by RNPs^20^. Nevertheless, Ll.LtrB function is inefficient in eurkaryotes, as it is limited by the low intracellular Mg^2+^ concentration, intron transcript NMD and translational repression^10^,^20^,^21^.

Future applications of group II introns for gene therapy and biotechnology would therefore require group II intron RNPs to enter the nucleus, to overcome these limitations. Studies of bacterial group II intron expression and localization in eukaryotic cells are therefore relevant. In yeast (*Saccharomyces cerevisiae*), the *L. lactis* Ll.LtrB intron-encoded protein (LtrA) has been reported to enter the nucleus after translation, provided that an SV-40 T-antigen nuclear localization signal (NLS)^10^ is added. LtrA also requires human codon use optimization for expression in mammalian cells. Previous studies in HEK-293 cells reported a homogeneous nuclear localization for the LtrA IEP, whereas the IEP was found to be excluded from the nucleolus in COS-7 cells, possibly due to aggregation resulting from the high level of protein production^22^.

We recently reported the expression and subcellular localization of the bacterial RmInt1 group II IEP, which lacks the endonuclease domain^23^, in *Arabidopsis thaliana* protoplasts^24^. This IEP has been shown to accumulate in the nucleolus, whereas a mutant IEP with a modified maturase domain was localized in nuclear speckles. The localization of the RmInt1 IEP in speckles in the absence of maturase activity is consistent with the hypothesis that group II introns are the ancestors of spliceosomal introns, because nuclear speckles are nuclear domains that accumulate high local concentrations of pre-mRNA splicing factors (snRNPs and SR proteins), located in the interchromatin nuclear space^25^.

We investigated the expression and subcellular distribution of the RmInt1 group II intron IEP in cultured human cells. We found that the wild-type RmInt1 IEP localized to the nucleus and nucleolus. Surprisingly, we also found that the IEP accumulated at the periphery of the nuclear matrix. Finally, we also observed coimmunoprecipitation of the RmInt1 spliced lariat with the IEP, suggesting that functional RNPs could be assembled in human cells.

## Results and Discussion

### Subcellular localization of the YFP-IEP RmIntI fusion in human cells

**Figure 1:**
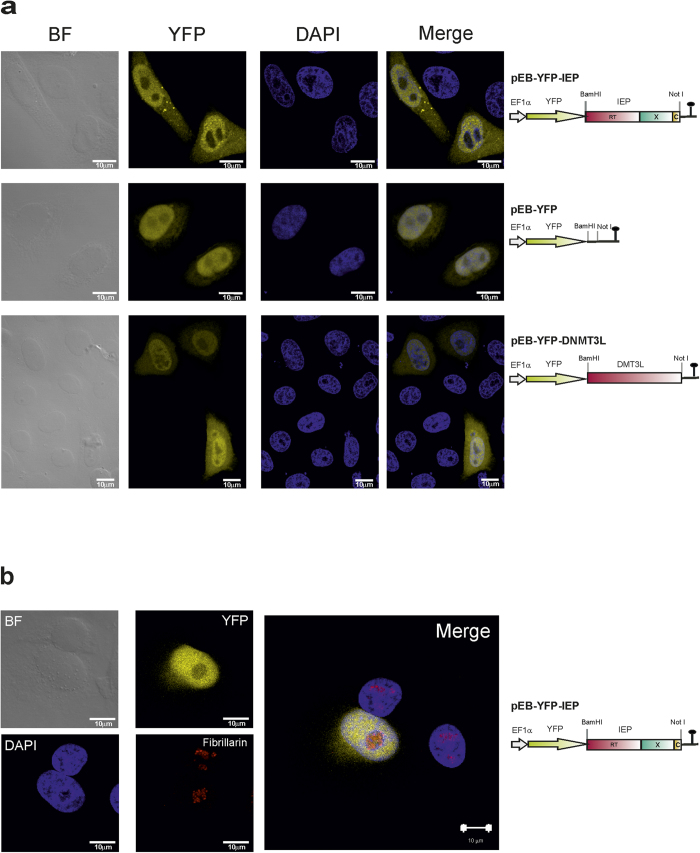
Subcellular distribution of the YFP-IEP RmInt1 fusion in HeLa cells. (**a**) Fluorescence and bright-field (BF) microscopy of transfected HeLa HA cells. Localization of the YFP-IEP fusion protein in yellow (YFP). DAPI (blue) was used to stain nuclear DNA, and merged images are shown in the right column. Schematic diagrams of the constructs are shown to the right of the micrographs. The domains of the IEP are indicated as follows: in pink, the reverse transcriptase; in green, the maturase and, in yellow, the C-terminal domain. (**b**) Colocalization experiments using an anti-fibrillarin antibody (red) as a nucleolar marker (Fibrillarin); The YFP-IEP fusion protein is shown in yellow (YFP); DAPI (blue) was used to stain nuclear DNA, and merged images are shown on the right. A diagram of the construct is shown to the right of the micrograph.

We investigated the intracellular distribution of the RmInt1 protein (IEP), by fusing its coding sequence to that of the yellow fluorescent protein (YFP), under control of the human EF1 α promoter (Pol II), in the pEB-YFP vector. We also generated two control vectors: pEB-YFP, encoding YFP only, and pEB-YFP DNMT3L, encoding YFP fused to the mouse *de novo* methyl transferase 3-like (DNMT3L) protein, a regulatory factor involved in DNA methylation^26^. In all constructs, the IEP and DNMT3L ORFs were fused to C-terminus of the YFP protein. Following the transfection of cultured human HeLa cells with these constructs, the YFP-IEP fusion protein was located in the nucleus but with some signal in the cytoplasm in 89.94% of the transfected cells. In 19.64% of the transfected cells, we also observed bright cytoplasmic foci (Fig. 1a). Remarkably, despite the localization of YFP-IEP to the nucleus, it appeared to be excluded from the nucleolus. This nucleolar exclusion was confirmed by colocalization experiments using the anti-fibrillarin antibody as a nucleolar marker (Fig. 1b). With the pEB-YFP and pEB-YFP-DNMT3L controls, preferential nuclear localization was observed for both the proteins encoded, but, by contrast to the YFP-IEP fusion, these proteins were also detected in the nucleolus (Fig. 1a). The cellular distribution of the IEP was unaffected in cells transfected with pEB-YFP-IEPΔORF, a construct that expresses the RmInt1 RNA together with the IEP, or with pEB-YFP-YYAAIEP, a construct encoding a mutated IEP with a modified maturase domain (residues Y354Y355 were replaced by alanine residues) (data not shown). Interestingly, the accumulation of the YFP-IEP fusion protein in the nucleus (74.5 kDa, thus exceeding the 40 kDa exclusion limit of the nuclear pore complex) suggests an active mechanism for translocation of the fusion protein through the nuclear membrane. Interestingly, LtrA, the protein encoded by the Ll.LtrB intron, requires the addition of a nuclear localization signal for translocation into the nucleus of human^22^ or yeast^10^ cells.

### Subcellular distribution of the FLAG-tagged RmInt1 IEP in human cells

**Figure 2:**
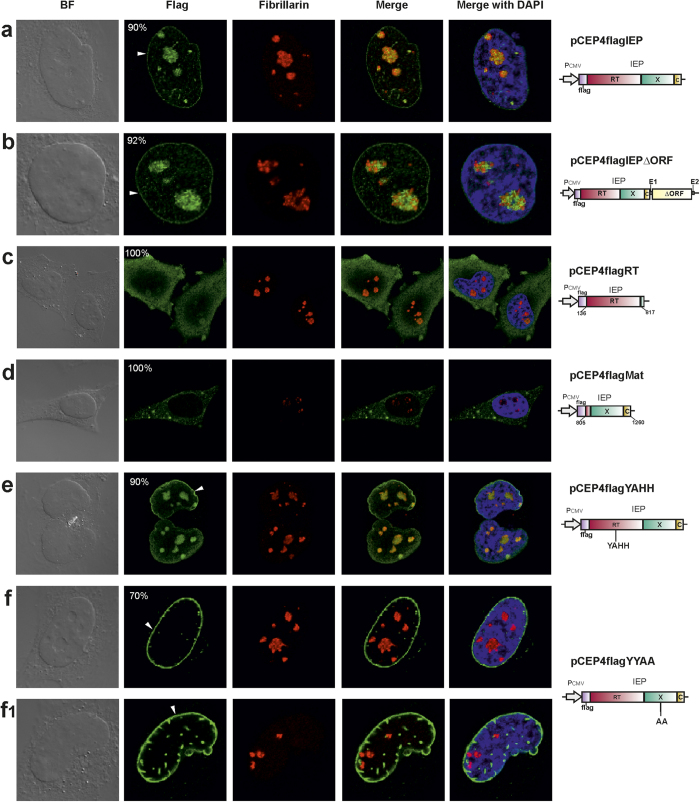
Subcellular distribution of FLAG-tagged IEP in HeLa cells. Immunofluorescence and bright-field (BF) microscopy of HeLa HA cells transfected with the constructs shown on the right. The domains of the IEP are indicated as follows: in pink, the reverse transcriptase; in green, the maturase and, in yellow, the C-terminal domain. Immunolocalization of FLAG-IEP, in green (Flag column). The nucleolus, detected with anti-fibrillarin antibody, is shown in red. A merged image of FLAG-IEP and the nucleolus is shown (merge column). DAPI (blue) was used to stain nuclear DNA. The numbers in the panels indicate the percentage of transfected cells displaying the corresponding fluorescence pattern. White arrowheads indicate signal surrounding the nuclear matrix.

For confirmation of the subcellular distribution of the RmInt1 IEP, and to check that there were no limitations associated with the use of YFP-fusion proteins, we then analyzed the subcellular distribution of FLAG-tagged IEP in cultured HeLa cells. We added a FLAG epitope to the N-terminus of the RmInt1 IEP and expressed the tagged-protein in the mammalian expression plasmid pCEP4, with expression under the control of the CMV (Pol II) promoter. As controls, we also generated N-terminal FLAG versions of mutated forms of the RmInt1 IEP. Following transfection, we found that the FLAG-tagged IEP was located in the nucleus, mostly in the nucleolus, but also at the periphery of the nuclear matrix, in 90% of the transfected cells (Fig. 2a). In the remaining 10% of the cells, the staining formed foci throughout the cell. Similar results were obtained with pCEP4flagIEPΔORF, in which the IEP was co-expressed with the RmInt1 ribozyme (Fig. 2b). The nucleolar localization of the FLAG-tagged IEP was further confirmed by colocalization experiments with an anti-fibrillarin antibody as a nucleolar marker. Thus, YFP seems to be primarily responsible for the exclusion of the IEP from the nucleolus. Furthermore, we speculate that localization to the periphery of the nuclear matrix may result from the direct association of the IEP with the inner nuclear membrane components or the nuclear lamina. The latter is absent from plant cells, potentially accounting for the distribution observed in *Arabidopsis* protoplasts, in which the RmInt1 IEP was also found in the nucleolus^24^, but not at the periphery of the nucleus.

We then investigated whether a specific region of the RmInt1 IEP was responsible for the observed localization to the nucleus and nucleolus, by tagging the maturase and RT segments of the IEP with a FLAG epitope. We found that 100% of the HeLa cells transfected with the construct encoding the FLAG-RT fusion (pCEP4flagRT) displayed cytoplasmic staining, with only a very weak signal in the nucleus and nucleolus (Fig. 2c), and no staining at the periphery of the nucleus. Furthermore, almost all the HeLa cells transfected with the construct encoding the FLAG-maturase fusion (pCEP4flagMat) displayed IEP accumulation in the cytoplasm, with a large number of cytoplasmic foci and no apparent signal in the nucleus or nucleolus (Fig. 2d). Overall, these results suggest that neither domain is itself sufficient to target the RmInt1 IEP to the nucleus. These findings contrast with those of protoplast studies, in which the maturase domain conferred localization to the nucleus, but not to the nucleolus. Thus, in mammalian cells, a complete functional protein seems to be required for the localization of the IEP to the nucleus.

We then investigated the effect of other mutations, affecting either RT or maturase activity, on the distribution of the RmInt1 IEP. We first analyzed the localization of a FLAG-IEP with an amino-acid substitution in the RT catalytic domain (YADD→YAHH [pCEP4flagYAHH]), resulting in a loss of RT activity, with the retention of about 80% of wild-type levels of splicing activity^27^. In 90% of the transfected cells, the distribution of the mutant IEP was identical to that of the wild-type IEP (Fig. 2e). In the remaining 10% of the cells, foci of staining were observed throughout the cell (data not shown). In transfected cells (Fig. 2f) expressing an IEP with an amino-acid substitution in the maturase domain (Y354Y355→AA) that abolished intron RNA splicing (pCEP4flagYYAA), the mutant IEP displayed strong localization to the periphery of the nucleus in 70% of the cells. In addition, 54% of these cells also displayed nuclear staining foci (Fig. 2f1). The IEP was not found in the nucleolus in any of the transfected cells. These results strongly suggest that nucleolar localization is linked to the maturase activity of the IEP or that the Y354Y355 amino acids are part of the signature responsible for targeting the IEP to the nucleolus.

### Expression of RmInt1ΔORF and IEP protein in human cells

**Figure 3:**
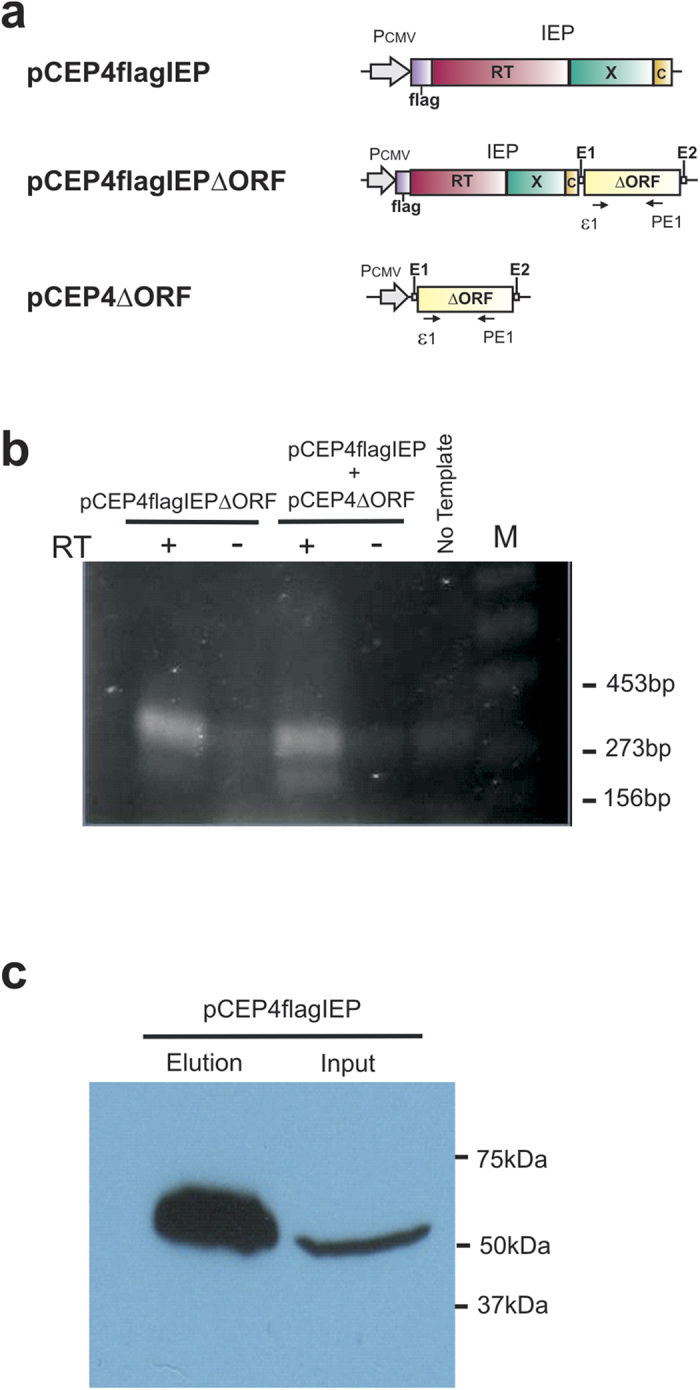
Detection of RmInt1 intron RNA and IEP in HeLa cells. (**a**) Diagram of the plasmids used in this study: each plasmid is a derivative of the pCEP4 episomal vector. The domains of the IEP are indicated as follows: in pink, the reverse transcriptase; in green, the maturase and, in yellow, the C-terminal domain. The positions of the primers used for cDNA synthesis (PE1), and for PCR amplification (ε1 and PE1) are also shown. (**b**) Detection of ΔORF RmInt1 *in vivo* by reverse transcription and PCR. PCR was carried out with (+) and without (−) prior reverse transcription (RT), with RNA from HeLa cells harboring pCEP4flagIEPΔORF or pCEP4flagIEP plus pCEP4ΔORF. The RT-PCR products were subjected to electrophoresis in a 2% agarose gel. M; molecular weight marker. (**c**) IEP protein detection in HeLa cells by SDS-PAGE and western blotting. Whole-cell extract (input) or immunoprecipitated (elution) products from cells harboring pCEP4flagIEP.

We studied the transcription of RmInt1 ΔORF (RmInt1 intron deleted from position 611 to 1,759), by transfecting HeLa cells with pCEP4flagIEPΔORF or cotransfecting them with pCEP4flagIEP and pCEP4ΔORF (Fig. 3a), and then subjecting the cells to selection on hygromycin for 10 days. RNA was then isolated and analyzed by RT-PCR (see Materials and Methods). The PCR products (Fig. 3b) were resolved by electrophoresis in 2% agarose gels. In both samples, amplification products of the expected size (~330 bp) were observed. These amplification products were not detected in samples to which no reverse transcriptase was added (RT-). For confirmation of the identity of the amplification products, the bands were excised from the gel, and the amplicons were cloned and analyzed by DNA sequencing. This analysis confirmed that the amplification products corresponded to the expected amplified fragments of the RmInt1 RNA. Thus, the RmInt1-derived intron ΔORF was expressed in the transfecting cells.

RmInt1 protein detection analyses were also carried out by immunoprecipitation in HeLa cells transfected with pCEP4flagIEP and selected on hygromycin for 10 days. Western blotting of the immunoprecipitated material with the anti-FLAG M2 antibody (Fig. 3c) revealed the presence of a 51.3 kDa band, corresponding to the expected size of the FLAG-IEP fusion. These results confirm that the RmInt1 IEP was produced in human cells.

### IEP and spliced RmInt1 RNA are co-immunoprecipitated

**Figure 4:**
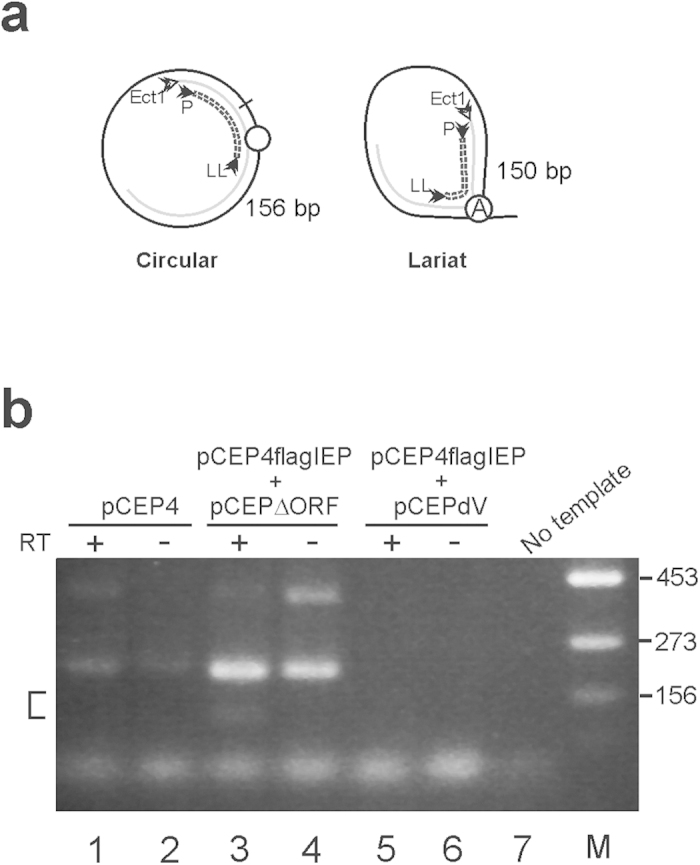
Identification of the spliced form of RmInt1 in FLAG-immunoprecipitate from transfected HeLa cell. (**a**) Representation of the PCR products obtained from the spliced intron as a circle or lariat. The circled A is the bulged adenosine residue in domain VI. (**b**) RT-PCR products were subjected to electrophoresis in a 2% agarose gel. PCR was performed with (+) or without (−) prior reverse transcription (RT), with the immunoprecipitates indicated. The area in which the DNA fragments were isolated is indicated by a square bracket. Other unspecific PCR products likely derived from contaminant genomic DNA was also observed. M: molecular weight marker; the numbers on the right indicate the size of the marker band in base pairs.

For confirmation of the physical association of the RmInt1 IEP with the spliced RNA of the RmInt1 intron, HeLa cells were transfected with pCEP4 or cotransfected with pCEP4flagIEP plus pCEP4ΔORF, or pCEP4flagIEP plus pCEP4dV (catalytic triad GTT is replaced by CGA in domain V of the RmInt1 ribozyme). Cell extracts were obtained and subjected to immunoprecipitation with the anti-FLAG M2 antibody fused to agarose beads, followed by western blotting, which demonstrated the presence of IEP in all samples, except those harboring pCEP4 alone (data not shown). The immunoprecipitated fractions were then used as a template for RT-PCR experiments, for detection of the spliced intron RNA. Amplification products were subjected to electrophoresis in 2% agarose gels (Fig. 4). Various bands were detected on the gel, and those of the expected size were isolated, cloned and sequenced. Only the immunoprecipitated material from samples cotransfected with pCEP4flagIEP plus pCEP4ΔORF contained the lariat (two of six clones) and circular (one of six clones) forms of the intron RNA. These data suggest that the IEP and intron RNA are associated and may be able to form a mature RNP.

## Conclusions

The group II intron RmInt1 IEP can be expressed under the control of a Pol II promoter, without codon optimization or a foreign nuclear localization signal, in human cells. It localizes to the nucleus and nucleolus in these cells, as well as the periphery of the nuclear matrix, where it may be associated with the nuclear lamina, which is absent in plant cells where the IEP was not located at the periphery of the nucleus^24^. In *A. thaliana* protoplasts the full-length IEP was found exclusively in the nucleolus, whereas the maturase domain alone targeted to nuclear speckles^24^. In human cells the maturase is not sufficient to target the RmInt1 IEP to the nucleus and a complete functional protein seems to be required. Nevertheless, our results strongly suggest that nucleolar localization in human cells is linked to the maturase activity of the IEP or that it contains amino acids that are part of the signature responsible for targeting the IEP to the nucleolus.

The intron RNA can also be expressed under the control of a Pol II promoter, in *cis* or in *trans,* without affecting the localization of the IEP. Furthermore, the RmInt1 RNA can also splice in human cells, generating intron lariat and circular forms that are co-immunoprecipitated with the IEP, probably forming mature RNPs. Note that we have detected spliced forms of RmInt1 in protoplasts, but all of them were derived from a wide range of processed forms, rather than from full-length intron lariat. Our results suggest that RmInt1 may be less subject to the limitations reported for group II intron Ll.LtrB in eukaryotes, including suboptimal codon usage, low Mg^2+^ concentration, translational repression and the NMD of intron RNA. RmInt1 may therefore be a group II intron suitable for use in gene targeting in higher organisms.

## Methods

### Strains and human cell lines

*Escherichia coli* DH5α was used for cloning and the maintenance of recombinant plasmids. It was grown overnight at 37 °C on Luria-Bertani (LB) medium. Antibiotics were added to the medium, when required, at the following concentrations: 200 μg/ml for ampicillin, and 10 μg/ml for tetracycline. Human HeLa-HA cells were maintained in an incubator (37 °C at a 7% CO_2_ level) and grown in minimum essential medium (GlutaMAX^TM^ supplement) (GIBCO-Invitrogen) supplemented with 10% heat-inactivated fetal bovine serum (GIBCO-Invitrogen), 1 x non-essential amino acids (GIBCO-Invitrogen). Cells were passaged by standard trypsin treatment, with a 0.05% stock solution (GIBCO-Invitrogen). The identity of the cell line was confirmed by STR analyses (Lorgen, Granada). We also used a PCR-based method (Minerva), to carry out routine tests for the absence of *Mycoplasma* spp.

### Plasmid constructs

pEB-YFP-D3L, encoding the human DNMT3L protein^26^, was digested with *Bam*HI and *Not*I, to excise the DNMT3L coding sequence and obtain the pEB-YFP plasmid. We inserted the IEP sequence amplified from pKGEMA4 with the YFP-IEP-5′ (5′-ACGGATCCACAAGTTTGTACAAAAAAGCAGGCTTCACTTCGGAAAGTA-3′) and YFP-IEP-3′ (5′-ACGCGGCCGCTCAGGTAAACGTGTTCGTTCCGA-3′) primers into this plasmid^27^. The IEP was cloned from the second codon (without the ATG) to generate the pEB-YFP-IEP construct. Similarly, pEB-YFP-IEPΔORF, a construct carrying the RmInt1 intron with a deletion from position 611 to 1,759, flanked by exon sequences -20/+5 and inserted into a *Bam*HI site just upstream from the IEP coding sequence^28^, was cloned after amplification with the YFP-IEP-5′ and YFP-ΔORF-3′ (5′-ACGCGGCCGCCTAGGCCAGGGGTGAGTAG-3′) primers, from the pKGEMA4 plasmid. Finally, a sequence encoding an IEP with an YY→AA substitution in the maturase domain (RmInt1 residues 354–355) was inserted to the pEB-YFP vector, using the *Bam*HI and *Not*I sites, after amplification from pKG2.5-A354A355 with the YFP-IEP-5′ and YFP-IEP-3′ primers^29^.

The constructs encoding the FLAG-tagged versions of the RmInt1 IEP were generated from the pCEP4 expression vector (Invitrogen) and contained the bacterial RmInt1 group II intron-encoded protein (IEP) sequence. The final expression vectors were obtained in the pICG^30^ vector, with a two-step-PCR, in which the first step was carried out with a Flag-LacZ-F oligomer (5′-GACTACAAAGACCATGACGGTGATTATAAAGATCATGACATCGATTACAAGGATGACGATGACAAGGATCCCGTCGTTTTACAACG-3′) and a LacZ-R oligomer (5′-CATTAAAGCGAGTGGCAACA-3′), and with the ICG-5 (5′-GAGCTCGAGATCTAGATATC-3′) and flag-CAT-R (5′-CTTGTCATCGTCATCCTTGTAATCGATGTCATGATCTTTATAATCACCGTCATGGTCTTTGTAGTCCATTTTAGCTTCCTTAGCTCCTG-3′) oligomers. In the second step, we combined the two PCR products and performed a third PCR with the LacZ-R and ICG-5 oligonucleotides. The resulting PCR products were inserted into pBluescript for sequencing, and then transferred to pICG digested with *Spe*I and *Bam*HI, to obtain the pICGflag plasmid. We introduced the Flag epitope sequence into this plasmid, downstream from the Psyn^31^ promoter. We used pKGEMA4 as a template, for PCR amplification of the IEP sequence with the IEP-BamHI-F (5′-ATGGATCCCATGACTTCGGAAA-3′) and IEP-BamHI-R (5′-GAGGATCCTCAGGTAAACGTGTT-3′) primers. We thus obtained the necessary amplicon to introducing an additional nucleotide to generate an in-frame N-terminal fusion to the Flag epitope. This fragment was inserted into the pICGflag vector to generate pICGflagIEP. The pICGflagIEPΔORF plasmid was generated in a similar manner, with the IEP-BamHI-F and ΔORF-BamHI-R (5′-ACGGATCCTAGGCCAGGGGTGAGTAG-3′) primers. The constructs for the expression and subcellular localization of the RmInt1 IEP based on pCEP4 were as follows. Use of the FwIEPNotI (5′-ACTCGCGGCCGCACCATGGACTACAAAGACCATGAC-3′) and RvIEPBglII (5′-ACTAGATCTTCAGGTAAACGTGTTCGTTC-3′) primers, with pICGflagIEP as the template, resulted in amplification of the FLAG-IEP fusion. This fragment was cloned in the pGEMT Easy vector system, to generate pGEflagIEP. This plasmid was digested with *Not*I, and the FLAG-IEP fusion sequence was inserted into the pCEP4 vector digested with the same enzyme to generate pCEP4flagIEP. Similarly, the pCEP4flagIEPΔORF vector and pCEP4flagRT (reverse transcriptase domain) were obtained with the primers FwIEPNotI and RvIEPΔORFBglII (5′-ACTAGATCTCCAGGGGTGAGTAGGCCGGA-3′) for pCEP4flagIEPΔORF, and FwIEPNotI and RvRT (5′-TATGCGGCCGCTCAGAAGAACTCGTCCCGCT-3′) for pCEP4flagRT. We used pICGflagIEPΔORF as a template for pCEP4flagIEPΔORF and pICGflagIEP as a template for pCEP4flagRT. The maturase domain (MAT) was amplified with primers FwMat (5′-TATCGGATCCCTACTGCAAGGATCAACGGCGCA-3′) and RvX (5′-TATCGGATCCTCAGGTAAACGTGTTCGTTCCGA-3′), from pICGflagIEP, used as a template. The maturase domain was also cloned in the pGEMT Easy vector system, to obtain pGEMat. The maturase domain was excised from this plasmid by *Bam*HI and inserted into pCEP4flagIEP digested with the same enzyme, to replace the IEP sequence with the maturase domain sequence and produce the pCEP4flagMat plasmid. Furthermore, a construct encoding Flag fused to an IEP carrying a RT domain with an amino-acid substitution (YADD→YAHH) was obtained by two-step PCR, in which primers containing the YAHH mutation were used, in the first step, to generate two partially overlapping PCR products. These two PCR products were mixed and amplified with external primers. The first two PCRs used pICGflagIEP plasmid as template. A protein carrying the YY→AA substitution in the maturase domain (RmIntI residues 354–355) was obtained in a similar manner. The sequences of the primers used were as follows: FwYAHH, (5′-CATGGTGTCGATATGCCCACCATGGTCTTGTTCA-3′), and RvYAHH, (5′-TGAACAAGACCATGGTGCGCATATCGACACCATG-3′) for YAHH; FwYYAA, (5′-GATGGATTGCCGCCGCGGGACGGTACAGTC-3′) and RvYYAA, (5′- CTACCTAACGGCGGCGCCCTGCCATGTCAG-3′) for YYAA. These fragments were inserted into the pGEM-T Easy vector to generate pGEflagYAHH and pGEflagYYAA. Both plasmids were digested with *Not*I, and the FlagYAHH fusion and the FlagYYAA fusion were inserted into pCEP4 digested with the same enzyme to generate pCEP4flagYAHH and pCEP4flagYYAA. The pCEP4-ΔORF construct was produced by inserting a PCR fragment containing ΔORF amplified with primers Reverse-ΔORF-BamHI and Forward-ΔORF-NotI between the *Not*I and *Bam*HI sites of pCEP4. pCEP4dV is a mutant with a defect of a splicing derivative of pCEP4-ΔORF, in which the catalytic triad GTT is replaced by CGA in domain V of the RmInt1 ribozyme^28^.

### Inmunocytochemistry

For subcellular localization of the RmInt1 FLAG-tagged IEP, HeLa cells were plated at a density of 10^5^ cells per well, in a six-well tissue culture plate. The following day, cells were transfected with 1 μg of purified DNA (Midiprep Plasmid DNA kit, QIAGEN), 3 μl of Xtreme 9 Transfection Reagent (Roche Applied Science) and 97 μl of room temperature (RT) warmed Optimen (Gibco, 31985-054) per well. The wells were treated with trypsin 24 h after transfection and the contents of each well were split into four samples, which were plated on sterile glass cover slips in 24-well tissue culture plates. The growth medium was removed 48 h after transfection, and the cells were fixed by incubation with 4% paraformaldehyde in 1 × PBS for 10 minutes. For analyses of the subcellular distribution of the YFP-IEP fusion proteins, HeLa cells were plated at a density of 2 × 10^4^ cells per well, in four-well Chamber Lab-Tek II slides (Thermo Scientific Nunc). The following day, cells were transfected with 0.5 μg of purified plasmid DNA (Midiprep Plasmid DNA Kit, QIAGEN), in the presence of 3 μl of Xtreme 9 Transfection Reagent. The growth medium was removed 24 h after transfection, and the cells were fixed by incubation for 10 minutes in 4% paraformaldehyde in 1 × PBS. In both cases, the cells were permeabilized by incubation with 0.1% Triton X-100 in 1 × PBS for 10 minutes, and nonspecific sites were saturated by incubation for 30 minutes with blocking buffer (10% goat serum [Abcam], 0.5% Triton X-100, 0.5% sodium azide in 1 × PBS). Cells were incubated overnight at 4 °C in hybridization solution (1% goat serum, 0.5% Triton X-100, 0.1% sodium azide in 1 × PBS) with a mouse anti-FLAG (1:500, F1804 Sigma-Aldrich), and/or rabbit anti-fibrillarin (Abcam). The next day the cells washed three times with 1% donkey serum in 1X PBS were incubated with secondary antibodies for 30 minutes. The following secondary antibodies were then added, in the same hybridization solution: Alexa Fluor 555 goat anti-mouse (1:1000 Life Technologies), and Alexa Fluor 488 donkey anti-rabbit (1:1000 Life Technologies) antibodies. The intrinsic fluorescent signal of the YFP-IEP proteins was used to determine their location within the cell. Then, cells were washed twice with 1% donkey serum in 1X PBS. For FLAG-tagged IEP, cells were mounted on a slide with Slowfade^®^ Gold Antifade Reagent and DAPI (Life Technologies). For YFP-IEP proteins, cells were mounted in Vectashield, with DAPI (Vector Laboratories). Analysis of YFP-IEP fusion protein was performed on 159 transfected cells. For FLAG-tagged IEP protein 100 cells were analyzed. Images were captured with a Zeiss LSM 710 confocal microscope. Finally, these images were processed with Zen 2009 Light Edition Software (Carl Zeiss Microimaging).

### Protein and RNA levels and western blot analysis

For analyses of protein and RNA levels, we plated 5 × 10^5^ HeLa HA cells in 100 mm dishes. The following day, the cells in each dish were transfected with 1 μg of plasmid DNA, prepared with a Midiprep Plasmid DNA kit (QIAGEN), in the presence of Xtreme 9 Transfection Reagent (Roche). Hygromycin (200 μg/ml) was added to the culture medium 48 hours after transfection, and the medium was replaced every other day. After 10 days of selection on hygromycin, the growth medium was removed from the cells. The cells were rinsed with 1 × PBS, collected with a cell scraper and spun at 2000 g during 5 minutes at 4 °C. RNA was isolated with the RNeasy Kit (Qiagen) according to the manufacturer’s instructions. RT-PCR was performed as previously described^32^. First-strand cDNA synthesis was performed with 1 μg of total RNA, 25 pmol of the PE1 specific primer (5′-TCCTCGACGCTCATACG-3′; complementary to nt 461-nt 477 sequence from the 5′ end of RmInt1) and superscript II RNase H^−^ reverse transcriptase (Invitrogen), as indicated in the manufacturer’s protocol. We used 1 μl of this reaction as a template for PCR, with 15 pmol of the PE1 primer and 15 pmol of the ε1 primer (5′-GTGAGCGTCGGATG-3′; complementary to the nt147-nt 164 sequence from the 5′ end of RmInt1). The PCR cycles were as follows: 94 °C for 3 minutes, followed by 35 cycles of 94 °C for 45 seconds, 55 °C for 30 seconds and 72 °C for 30 seconds. A final extension was then carried out at 72 °C for 10 minutes. The RT-PCR products (20 μl) were resolved by electrophoresis in a 2% agarose gel. The DNA fragments of the expected size were isolated from the gel with the IllustraTM GFX^TM^ PCR DNA and gel band purification kit (GE Healthcare) and the samples were sequenced.

Inmunoprecipitation was carried out with the tagged protein inmunoprecipitation kit (Sigma). We added 1x Complete Mini EDTA-free protease Inhibitor Cocktail (Roche Applied Science) and used the Bio-Rad Protein Assay (Bio-Rad) to determine protein concentration. The total amount of protein present, after lysis, was 1.24 mg/ml per sample. The immunoprecipitated proteins were resolved by electrophoresis in a denaturing 10% polyacrylamide gel containing SDS and were transferred to nitrocellulose membranes by electroblotting for 50 minutes at 50 mA (TE77PWR semidry apparatus, Amersham Biosciences). All of the post-elution protein obtained (3 μg) was loaded in a single lane. Another lane was loaded with 3.7 μg of whole-cell extract (input). Western blotting was carried out using anti-FLAG antibody (Sigma-Aldrich F1804), rabbit anti-mouse HRP-conjugated secondary antibody (Sigma-Aldrich A9044) and was developed using Amersham ECL Advance Western Blotting detection kit (GE Healthcare), and the blot was placed against Konica Minolta medical film.

RT-PCR was performed on immunoprecipitated fractions as previously described^32^, with 10 μl of the immunoprecipitate to be reverse transcribed and 25 pmol of the Ect1 primer (5′-CACCTGCTCGGATCTCGTC-3′). PCR was carried out with 2 μl of cDNA as the template, and the primers P (5′-TGAAAGCCGATCCCGGAG-3′) and LL (5′-GAGGTTCACGCACCGTTCTG-3′; complementary to a sequence 59–49 nt from the 3′ end of RmInt1). We ran 10 μl of the RT-PCR products on a 2% agarose gel and DNA fragments of the expected size were isolated from the gel with the Illustra^TM^ GFX^TM^ PCR DNA and gel band purification kit (GE Healthcare). The DNA was inserted into the pGEMT-easy vector (Promega) and sequenced.

## Additional Information

**How to cite this article**: Reinoso-Colacio, M. *et al.* Localization of a bacterial group II intron-encoded protein in human cells. *Sci. Rep.* **5**, 12716; doi: 10.1038/srep12716 (2015).
